# Crystal structure of hexa­prop-2-en-1-yl 4,4′,4′′,4′′′,4′′′′,4′′′′′-[1,3,5,2λ^5^,4λ^5^,6λ^5^-tri­aza­triphosphinine-2,2,4,4,6,6-hexa­yl­hexa­kis­(­oxy)]hexa­benzoate

**DOI:** 10.1107/S2056989015021301

**Published:** 2015-11-18

**Authors:** Jing Zhu, Qian Li, Fu-Wei Zheng, Juan He, Ling-Bo Qu

**Affiliations:** a College of Chemistry and Chemical Engineering, Henan University of Technology, Zhengzhou 450001, People’s Republic of China

**Keywords:** crystal structure, cyclo­tri­aza­triphosphinine, organic-inorganic compounds

## Abstract

In the title compound, C_60_H_54_N_3_O_18_P_3_, the central phosphazene ring is essentially planar, with an r.m.s. deviation of the six fitted atoms of 0.068 Å. The P—N bond lengths are within the narrow range 1.575 (2)–1.585 (2) Å, indicating the electrons are delocalized within the ring. The two ethenyl benzoate substituents on each P atom are located up and down with respect to the plane of the central P_3_N_3_ ring. The atoms of two terminal propenyl groups are disordered over two sets of sites, with refined site-occupancy ratios of 0.249 (12):0.751 (12) and 0.476 (9):0.524 (9). No intermolecular interactions are observed.

## Related literature   

Cyclo­triphosphazene derivatives feature a planar six-membered ring consisting of alternating N and P atoms (Wu *et al.*, 2011 ▸). Their potential applications include solid polymer electrolytes (Allcock *et al.*, 2001 ▸; Chen-Yang *et al.*, 2000 ▸), flame retardants (Levchik *et al.*, 2000 ▸), non-linear optics (Rojo *et al.*, 2000 ▸) and biodegradable materials (Ibim *et al.*, 1997 ▸). The title compound was prepared according to a literature procedure (Guo *et al.*, 2009 ▸).
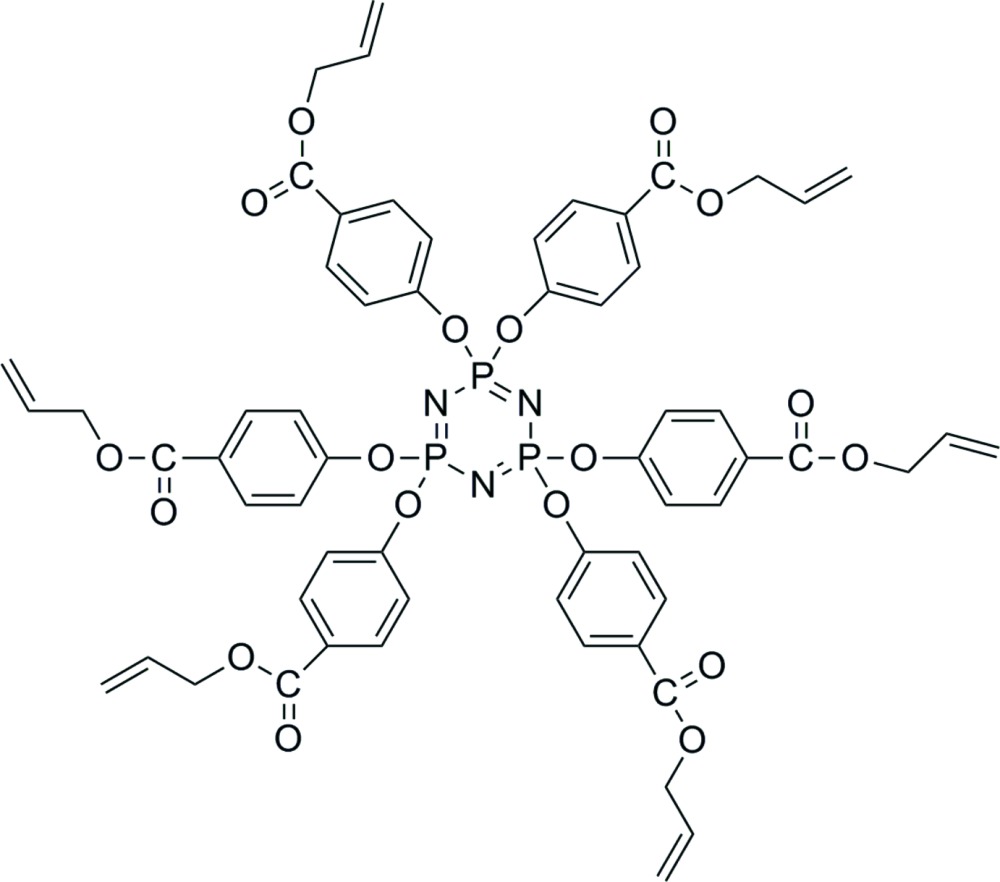


## Experimental   

### Crystal data  

C_60_H_54_N_3_O_18_P_3_
*M* *_r_* = 1197.97
Monoclinic, 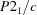

*a* = 7.97548 (11) Å
*b* = 16.9389 (3) Å
*c* = 43.0661 (7) Å
β = 93.6340 (14)°
*V* = 5806.36 (16) Å^3^
*Z* = 4
Cu *K*α radiation
μ = 1.59 mm^−1^
*T* = 291 K
0.22 × 0.16 × 0.15 mm

### Data collection  

Agilent Xcalibur Eos Gemini diffractometer
Absorption correction: multi-scan (*CrysAlis PRO*; Agilent, 2011 ▸) *T*_min_ = 0.664, *T*_max_ = 1.000
21543 measured reflections
10382 independent reflections
7375 reflections with *I* > 2σ(*I*)
*R*_int_ = 0.027

### Refinement  

*R*[*F* ^2^ > 2σ(*F* ^2^)] = 0.052
*wR*(*F* ^2^) = 0.150
*S* = 1.03
10382 reflections
774 parameters
11 restraints
H-atom parameters constrained
Δρ_max_ = 0.28 e Å^−3^
Δρ_min_ = −0.30 e Å^−3^

### 

Data collection: *CrysAlis PRO* (Agilent, 2011 ▸); cell refinement: *CrysAlis PRO*; data reduction: *CrysAlis PRO*; program(s) used to solve structure: *SHELXS97* (Sheldrick, 2008 ▸); program(s) used to refine structure: *SHELXL97* (Sheldrick, 2008 ▸); molecular graphics: *OLEX2* (Dolomanov *et al.*, 2009 ▸); software used to prepare material for publication: *OLEX2*.

## Supplementary Material

Crystal structure: contains datablock(s) I. DOI: 10.1107/S2056989015021301/zq2233sup1.cif

Structure factors: contains datablock(s) I. DOI: 10.1107/S2056989015021301/zq2233Isup2.hkl

Supporting information file. DOI: 10.1107/S2056989015021301/zq2233Isup3.cml

**Fig. 1.**
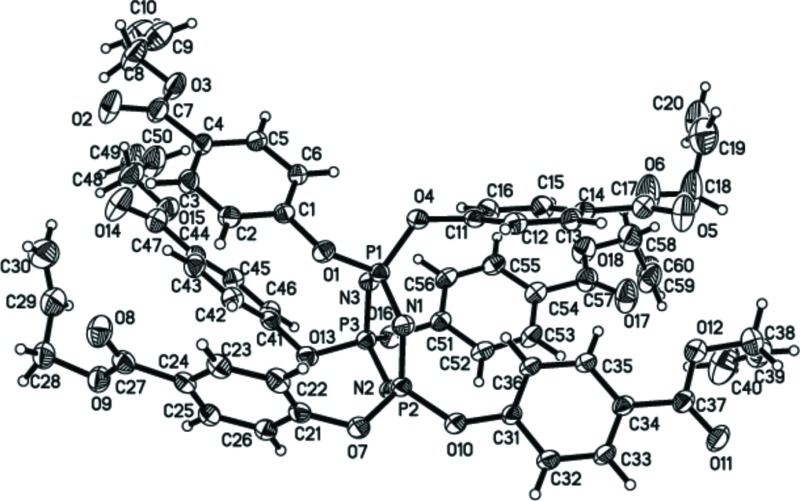
The molecular structure of the title compound showing 50% probability displacement ellipsoids (some disordered parts are omitted for clarity).

. DOI: 10.1107/S2056989015021301/zq2233fig1.tif
The mol­ecular structure of the title compound showing 50% probability displacement ellipsoids (some disordered parts are omitted for clarity).

CCDC reference: 1436029

Additional supporting information:  crystallographic information; 3D view; checkCIF report

## Figures and Tables

**Table 1:** Selected bond lengths (Å)

N1—P1	1.580 (2)
N1—P2	1.582 (2)
N2—P2	1.575 (2)
N2—P3	1.579 (2)
N3—P1	1.578 (2)
N3—P3	1.585 (2)

## supplementary crystallographic information

### S1. Comment

Cyclotriphosphazene derivatives are typical classes of organic-inorganic compounds with a planar six-membered ring consisting of alternating N and P atoms (Wu *et al.*, 2011). Their potential applications include solid polymer electrolytes (Allcock *et al.*, 2001; Chen-Yang *et al.*, 2000), flame retardants (Levchik *et al.*, 2000), nonlinear optics (Rojo *et al.*, 2000) and biodegradable materials (Ibim *et al.*, 1997). The title compound was prepared according to a literature report (Guo *et al.*, 2009).

### S2. Experimental

A mixture of hexachlorocyclotriphosphazene (1.04 g, 3 mmol), allyl 4-hydroxybenzoate (3.75 g, 21 mmol), and activated potassium carbonate (3.5 g, 253 mmol) in tetrahydrofuran (50 ml) was stirred at 66 °C for 45 h under nitrogen. The resulting suspension mixture was filtered and the filtrate was concentrated, leading to the formation of a faint yellow viscous liquid. It was dissolve in 20 ml ethyl acetate, and the solution was added dropwise to methanol. Colourless needle crystals suitable for X-ray diffraction analysis were obtained by slow evaporation of the solvent after about 2 days.

### S3. Refinement

Crystal data, data collection and structure refinement details are summarized in Table 1. Hydrogen atoms were placed and refined at idealized positions riding on the carbon atoms with isotropic displacement parameters *U*_iso_(H) = 1.2 *U*_eq_(C) and aromatic C–H = 0.93 Å and methylene C–H = 0.97 Å. The atoms of two terminal propenyl groups are disordered over two sets of sites with refined site-occupancy ratios of 0.249 (12):0.751 (12) and 0.476 (9):0.524 (9). Some restraints were used to correct the geometry and the thermal parameters of the terminal propenyl ligands and the corresponding atoms.

### Crystal data

**Table:**

C_60_H_54_N_3_O_18_P_3_	*F*(000) = 2496
*M**_r_* = 1197.97	*D*_x_ = 1.370 Mg m^−^^3^
Monoclinic, *P*2_1_/*c*	Cu *K*α radiation, λ = 1.54184 Å
*a* = 7.97548 (11) Å	Cell parameters from 6834 reflections
*b* = 16.9389 (3) Å	θ = 4.1–72.3°
*c* = 43.0661 (7) Å	µ = 1.59 mm^−^^1^
β = 93.6340 (14)°	*T* = 291 K
*V* = 5806.36 (16) Å^3^	Block, colourless
*Z* = 4	0.22 × 0.16 × 0.15 mm

### Data collection

**Table:**

Agilent Xcalibur Eos Gemini diffractometer	10382 independent reflections
Radiation source: Enhance (Cu) X-ray Source	7375 reflections with *I* > 2σ(*I*)
Graphite monochromator	*R*_int_ = 0.027
Detector resolution: 16.2312 pixels mm^-1^	θ_max_ = 67.1°, θ_min_ = 3.3°
ω scans	*h* = −9→7
Absorption correction: multi-scan (*CrysAlis PRO*; Agilent, 2011)	*k* = −20→15
*T*_min_ = 0.664, *T*_max_ = 1.000	*l* = −51→50
21543 measured reflections	

### Refinement

**Table:**

Refinement on *F*^2^	Primary atom site location: structure-invariant direct methods
Least-squares matrix: full	Hydrogen site location: inferred from neighbouring sites
*R*[*F*^2^ > 2σ(*F*^2^)] = 0.052	H-atom parameters constrained
*wR*(*F*^2^) = 0.150	*w* = 1/[σ^2^(*F*_o_^2^) + (0.078*P*)^2^ + 0.6732*P*] where *P* = (*F*_o_^2^ + 2*F*_c_^2^)/3
*S* = 1.03	(Δ/σ)_max_ < 0.001
10382 reflections	Δρ_max_ = 0.28 e Å^−^^3^
774 parameters	Δρ_min_ = −0.30 e Å^−^^3^
11 restraints	

### Special details

**Table:**

Geometry. All e.s.d.'s (except the e.s.d. in the dihedral angle between two l.s. planes) are estimated using the full covariance matrix. The cell e.s.d.'s are taken into account individually in the estimation of e.s.d.'s in distances, angles and torsion angles; correlations between e.s.d.'s in cell parameters are only used when they are defined by crystal symmetry. An approximate (isotropic) treatment of cell e.s.d.'s is used for estimating e.s.d.'s involving l.s. planes.

### Fractional atomic coordinates and isotropic or equivalent isotropic displacement parameters (Å^2^)

**Table:**

	*x*	*y*	*z*	*U*_iso_*/*U*_eq_	Occ. (<1)
C1	0.6244 (3)	0.11071 (16)	0.40893 (6)	0.0541 (6)	
C2	0.6114 (3)	0.10471 (19)	0.44061 (6)	0.0642 (7)	
H2	0.5664	0.0596	0.4491	0.077*	
C3	0.6654 (4)	0.1660 (2)	0.45932 (6)	0.0696 (8)	
H3	0.6579	0.1621	0.4807	0.084*	
C4	0.7309 (3)	0.23365 (19)	0.44693 (6)	0.0645 (7)	
C5	0.7474 (3)	0.23766 (18)	0.41498 (6)	0.0626 (7)	
H5	0.7942	0.2824	0.4065	0.075*	
C6	0.6950 (3)	0.17606 (17)	0.39577 (6)	0.0591 (6)	
H6	0.7071	0.1785	0.3745	0.071*	
C7	0.7807 (4)	0.3006 (2)	0.46779 (7)	0.0805 (9)	
C8	0.8574 (8)	0.4358 (3)	0.47158 (11)	0.1331 (19)	
H8A	0.7679	0.4474	0.4851	0.160*	
H8B	0.9595	0.4260	0.4844	0.160*	
C9	0.8811 (7)	0.5006 (3)	0.45159 (14)	0.1336 (18)	
H9	0.9405	0.4907	0.4341	0.160*	
C10	0.8301 (8)	0.5704 (4)	0.45499 (16)	0.164 (2)	
H10A	0.7701	0.5834	0.4721	0.196*	
H10B	0.8526	0.6088	0.4404	0.196*	
C11	0.6976 (3)	0.03458 (16)	0.30966 (6)	0.0570 (6)	
C12	0.7519 (3)	−0.03787 (17)	0.29997 (7)	0.0674 (7)	
H12	0.7694	−0.0791	0.3140	0.081*	
C13	0.7798 (4)	−0.04811 (18)	0.26889 (7)	0.0684 (7)	
H13	0.8159	−0.0969	0.2620	0.082*	
C14	0.7551 (3)	0.01275 (18)	0.24782 (7)	0.0634 (7)	
C15	0.6996 (4)	0.08501 (18)	0.25824 (8)	0.0726 (8)	
H15	0.6810	0.1263	0.2442	0.087*	
C16	0.6717 (4)	0.09615 (17)	0.28926 (8)	0.0706 (8)	
H16	0.6357	0.1449	0.2962	0.085*	
C17	0.7906 (4)	−0.0018 (2)	0.21482 (8)	0.0823 (9)	
C18	0.778 (7)	0.075 (3)	0.1662 (13)	0.187 (6)	0.249 (12)
H18A	0.6815	0.1066	0.1594	0.225*	0.249 (12)
H18B	0.7692	0.0246	0.1553	0.225*	0.249 (12)
C18A	0.824 (3)	0.0479 (6)	0.1652 (4)	0.187 (6)	0.751 (12)
H18C	0.9116	0.0084	0.1648	0.225*	0.751 (12)
H18D	0.7269	0.0294	0.1525	0.225*	0.751 (12)
C19	0.940 (6)	0.1168 (15)	0.1587 (9)	0.158 (4)	0.249 (12)
H19	1.0426	0.0911	0.1625	0.189*	0.249 (12)
C19A	0.8830 (17)	0.1235 (7)	0.1533 (3)	0.158 (4)	0.751 (12)
H19A	0.9344	0.1218	0.1346	0.189*	0.751 (12)
C20	0.939 (5)	0.1908 (17)	0.1466 (6)	0.171 (4)	0.249 (12)
H20C	0.8373	0.2172	0.1426	0.205*	0.249 (12)
H20D	1.0391	0.2152	0.1422	0.205*	0.249 (12)
C20A	0.8722 (14)	0.1868 (6)	0.1651 (2)	0.171 (4)	0.751 (12)
H20A	0.8219	0.1919	0.1839	0.205*	0.751 (12)
H20B	0.9143	0.2312	0.1555	0.205*	0.751 (12)
C21	0.1451 (4)	−0.05609 (17)	0.39968 (7)	0.0636 (7)	
C22	0.2751 (4)	−0.07835 (17)	0.42059 (7)	0.0692 (7)	
H22	0.3548	−0.1148	0.4149	0.083*	
C23	0.2849 (4)	−0.04589 (17)	0.44979 (7)	0.0674 (7)	
H23	0.3700	−0.0615	0.4642	0.081*	
C24	0.1684 (3)	0.01032 (17)	0.45792 (6)	0.0603 (6)	
C25	0.0372 (3)	0.03084 (18)	0.43663 (7)	0.0662 (7)	
H25	−0.0429	0.0673	0.4422	0.079*	
C26	0.0250 (3)	−0.00250 (19)	0.40739 (7)	0.0666 (7)	
H26	−0.0628	0.0110	0.3931	0.080*	
C27	0.1922 (4)	0.04851 (19)	0.48899 (7)	0.0687 (7)	
C28	0.1005 (5)	0.1519 (2)	0.52103 (8)	0.0896 (10)	
H28A	0.1538	0.1212	0.5379	0.107*	
H28B	−0.0070	0.1705	0.5275	0.107*	
C29	0.2074 (7)	0.2197 (3)	0.51407 (10)	0.1106 (13)	
H29	0.1581	0.2576	0.5008	0.133*	
C30	0.3543 (7)	0.2326 (4)	0.52357 (13)	0.146 (2)	
H30A	0.4107	0.1969	0.5369	0.175*	
H30B	0.4085	0.2781	0.5174	0.175*	
C31	0.2102 (3)	−0.12102 (16)	0.28705 (7)	0.0602 (6)	
C32	0.1303 (4)	−0.18309 (18)	0.27119 (8)	0.0712 (8)	
H32	0.0567	−0.2158	0.2811	0.085*	
C33	0.1619 (4)	−0.19549 (18)	0.24055 (7)	0.0712 (8)	
H33	0.1093	−0.2371	0.2297	0.085*	
C34	0.2695 (3)	−0.14752 (17)	0.22575 (7)	0.0613 (7)	
C35	0.3457 (3)	−0.08538 (18)	0.24187 (7)	0.0660 (7)	
H35	0.4174	−0.0520	0.2318	0.079*	
C36	0.3169 (3)	−0.07213 (18)	0.27280 (7)	0.0672 (7)	
H36	0.3695	−0.0306	0.2836	0.081*	
C37	0.3047 (4)	−0.16567 (19)	0.19295 (7)	0.0693 (8)	
C38	0.4739 (6)	−0.1337 (3)	0.15172 (10)	0.1140 (15)	
H38A	0.4526	−0.1885	0.1462	0.137*	
H38B	0.5935	−0.1242	0.1509	0.137*	
C39	0.3816 (9)	−0.0826 (4)	0.12904 (12)	0.135 (2)	
H39	0.4000	−0.0918	0.1082	0.162*	
C40	0.2810 (12)	−0.0279 (4)	0.13427 (17)	0.186 (3)	
H40A	0.2576	−0.0159	0.1546	0.223*	
H40B	0.2299	0.0006	0.1178	0.223*	
C41	0.1543 (3)	0.22081 (17)	0.38323 (6)	0.0589 (6)	
C42	0.2401 (4)	0.20936 (19)	0.41142 (7)	0.0709 (8)	
H42	0.2662	0.1587	0.4185	0.085*	
C43	0.2871 (4)	0.2743 (2)	0.42918 (7)	0.0761 (8)	
H43	0.3449	0.2672	0.4484	0.091*	
C44	0.2497 (4)	0.3495 (2)	0.41884 (7)	0.0694 (7)	
C45	0.1597 (4)	0.35902 (19)	0.39029 (7)	0.0720 (8)	
H45	0.1324	0.4095	0.3832	0.086*	
C46	0.1106 (4)	0.29416 (18)	0.37242 (7)	0.0672 (7)	
H46	0.0493	0.3004	0.3535	0.081*	
C47	0.3088 (5)	0.4170 (2)	0.43880 (9)	0.0899 (10)	
C48	0.3453 (7)	0.5550 (3)	0.44269 (12)	0.1274 (17)	
H48A	0.2697	0.5651	0.4590	0.153*	
H48B	0.4571	0.5459	0.4522	0.153*	
C49	0.3467 (9)	0.6198 (3)	0.42206 (13)	0.159 (3)	
H49	0.4005	0.6126	0.4037	0.191*	
C50	0.2793 (10)	0.6890 (3)	0.42633 (14)	0.187 (3)	
H50A	0.2242	0.6990	0.4443	0.225*	
H50B	0.2866	0.7283	0.4114	0.225*	
C51	0.2073 (3)	0.17633 (17)	0.28273 (6)	0.0604 (7)	
C52	0.1644 (4)	0.11589 (19)	0.26258 (7)	0.0733 (8)	
H52	0.1011	0.0734	0.2689	0.088*	
C53	0.2171 (4)	0.11929 (19)	0.23266 (7)	0.0783 (9)	
H53	0.1907	0.0782	0.2189	0.094*	
C54	0.3091 (4)	0.18341 (18)	0.22297 (7)	0.0694 (7)	
C55	0.3487 (4)	0.24375 (18)	0.24349 (7)	0.0725 (8)	
H55	0.4094	0.2871	0.2371	0.087*	
C56	0.2988 (4)	0.24022 (17)	0.27351 (7)	0.0700 (8)	
H56	0.3267	0.2807	0.2875	0.084*	
C57	0.3593 (5)	0.1855 (2)	0.19026 (9)	0.0864 (9)	
C58	0.4440 (7)	0.2653 (3)	0.14879 (10)	0.1182 (15)	
H58A	0.4978	0.3161	0.1463	0.142*	0.524 (9)
H58B	0.5242	0.2246	0.1441	0.142*	0.524 (9)
H58C	0.4716	0.2142	0.1403	0.142*	0.476 (9)
H58D	0.5412	0.2994	0.1478	0.142*	0.476 (9)
C59	0.300 (2)	0.2597 (7)	0.1268 (3)	0.129 (3)	0.524 (9)
H59	0.2391	0.2131	0.1237	0.155*	0.524 (9)
C59A	0.298 (3)	0.3000 (9)	0.1303 (4)	0.129 (3)	0.476 (9)
H59A	0.2573	0.3477	0.1374	0.155*	0.476 (9)
C60	0.260 (2)	0.3244 (8)	0.1116 (3)	0.145 (4)	0.524 (9)
H60A	0.3229	0.3700	0.1152	0.175*	0.524 (9)
H60B	0.1682	0.3250	0.0971	0.175*	0.524 (9)
C60A	0.222 (2)	0.2706 (10)	0.1051 (3)	0.145 (4)	0.476 (9)
H60C	0.2580	0.2231	0.0971	0.175*	0.476 (9)
H60D	0.1314	0.2973	0.0953	0.175*	0.476 (9)
N1	0.4142 (3)	−0.03827 (13)	0.34966 (6)	0.0611 (5)	
N2	0.1214 (3)	0.03232 (14)	0.33232 (6)	0.0646 (6)	
N3	0.3977 (2)	0.12059 (12)	0.34622 (5)	0.0525 (5)	
O1	0.5647 (2)	0.04576 (11)	0.39149 (4)	0.0623 (5)	
O2	0.7857 (4)	0.29681 (18)	0.49581 (6)	0.1182 (10)	
O3	0.8136 (4)	0.36594 (16)	0.45250 (5)	0.0978 (8)	
O4	0.6794 (2)	0.04569 (12)	0.34135 (4)	0.0628 (5)	
O5	0.8288 (5)	−0.0643 (2)	0.20453 (7)	0.1317 (11)	
O6	0.7770 (6)	0.0616 (2)	0.19797 (7)	0.1519 (15)	
O7	0.1338 (3)	−0.08998 (12)	0.36977 (5)	0.0729 (5)	
O8	0.3057 (3)	0.03484 (16)	0.50763 (5)	0.0985 (8)	
O9	0.0748 (3)	0.10293 (15)	0.49350 (5)	0.0820 (6)	
O10	0.1743 (3)	−0.11468 (12)	0.31813 (5)	0.0729 (5)	
O11	0.2382 (3)	−0.21739 (15)	0.17770 (5)	0.0927 (7)	
O12	0.4256 (3)	−0.11996 (16)	0.18287 (5)	0.0939 (7)	
O13	0.1013 (2)	0.15318 (12)	0.36631 (5)	0.0672 (5)	
O14	0.3671 (5)	0.4108 (2)	0.46479 (8)	0.1449 (13)	
O15	0.2883 (4)	0.48591 (16)	0.42427 (6)	0.0997 (8)	
O16	0.1472 (2)	0.17717 (12)	0.31240 (5)	0.0684 (5)	
O17	0.3589 (5)	0.12874 (18)	0.17373 (7)	0.1382 (13)	
O18	0.4004 (4)	0.25671 (15)	0.18104 (6)	0.1042 (8)	
P1	0.50200 (8)	0.04433 (4)	0.35587 (2)	0.05211 (17)	
P2	0.21804 (8)	−0.04575 (4)	0.34213 (2)	0.05849 (18)	
P3	0.20045 (8)	0.11649 (4)	0.33920 (2)	0.05513 (18)	

### Atomic displacement parameters (Å^2^)

**Table:**

	*U*^11^	*U*^22^	*U*^33^	*U*^12^	*U*^13^	*U*^23^
C1	0.0474 (13)	0.0592 (15)	0.0551 (14)	0.0035 (11)	−0.0022 (10)	0.0019 (12)
C2	0.0604 (15)	0.0723 (18)	0.0592 (15)	−0.0039 (13)	−0.0004 (12)	0.0131 (14)
C3	0.0712 (17)	0.088 (2)	0.0489 (14)	0.0054 (16)	−0.0019 (12)	0.0040 (14)
C4	0.0612 (15)	0.0780 (19)	0.0534 (15)	0.0001 (14)	−0.0039 (12)	−0.0053 (14)
C5	0.0603 (15)	0.0688 (17)	0.0584 (15)	−0.0101 (13)	0.0015 (12)	−0.0002 (13)
C6	0.0587 (14)	0.0692 (17)	0.0495 (13)	−0.0047 (13)	0.0051 (11)	0.0000 (12)
C7	0.086 (2)	0.092 (2)	0.0621 (19)	−0.0028 (18)	−0.0086 (15)	−0.0119 (17)
C8	0.196 (5)	0.106 (3)	0.092 (3)	−0.048 (3)	−0.032 (3)	−0.024 (3)
C9	0.154 (5)	0.100 (4)	0.142 (4)	−0.020 (3)	−0.029 (3)	−0.034 (3)
C10	0.186 (6)	0.114 (4)	0.184 (6)	0.000 (4)	−0.040 (5)	−0.042 (4)
C11	0.0389 (12)	0.0616 (16)	0.0707 (16)	−0.0037 (11)	0.0051 (11)	−0.0085 (13)
C12	0.0586 (15)	0.0586 (16)	0.084 (2)	0.0042 (13)	0.0009 (13)	−0.0022 (14)
C13	0.0608 (16)	0.0594 (17)	0.085 (2)	0.0078 (13)	0.0034 (14)	−0.0188 (15)
C14	0.0495 (14)	0.0664 (17)	0.0746 (17)	−0.0072 (12)	0.0059 (12)	−0.0117 (15)
C15	0.079 (2)	0.0592 (17)	0.080 (2)	0.0002 (15)	0.0150 (15)	0.0006 (15)
C16	0.0751 (19)	0.0506 (15)	0.088 (2)	0.0061 (13)	0.0166 (15)	−0.0078 (15)
C17	0.081 (2)	0.088 (2)	0.079 (2)	−0.0108 (18)	0.0066 (16)	−0.015 (2)
C18	0.380 (18)	0.108 (9)	0.081 (3)	0.001 (9)	0.069 (7)	−0.009 (7)
C18A	0.380 (18)	0.108 (9)	0.081 (3)	0.001 (9)	0.069 (7)	−0.009 (7)
C19	0.197 (12)	0.185 (8)	0.090 (6)	0.028 (7)	−0.006 (6)	0.017 (6)
C19A	0.197 (12)	0.185 (8)	0.090 (6)	0.028 (7)	−0.006 (6)	0.017 (6)
C20	0.258 (11)	0.148 (7)	0.109 (6)	0.018 (7)	0.038 (6)	−0.016 (6)
C20A	0.258 (11)	0.148 (7)	0.109 (6)	0.018 (7)	0.038 (6)	−0.016 (6)
C21	0.0635 (16)	0.0571 (16)	0.0716 (17)	−0.0125 (13)	0.0159 (13)	−0.0035 (13)
C22	0.0723 (18)	0.0559 (16)	0.0813 (19)	0.0079 (14)	0.0197 (15)	0.0031 (14)
C23	0.0669 (17)	0.0659 (17)	0.0702 (17)	0.0072 (14)	0.0102 (13)	0.0164 (14)
C24	0.0591 (15)	0.0614 (16)	0.0617 (15)	−0.0038 (12)	0.0144 (12)	0.0077 (13)
C25	0.0560 (15)	0.0740 (19)	0.0693 (17)	0.0030 (13)	0.0108 (12)	−0.0036 (14)
C26	0.0519 (14)	0.0763 (19)	0.0719 (17)	−0.0005 (13)	0.0056 (12)	−0.0075 (15)
C27	0.0709 (18)	0.0712 (19)	0.0650 (17)	−0.0030 (15)	0.0114 (14)	0.0125 (14)
C28	0.104 (3)	0.091 (3)	0.074 (2)	−0.004 (2)	0.0143 (18)	−0.0147 (19)
C29	0.125 (4)	0.107 (3)	0.100 (3)	−0.008 (3)	0.009 (3)	−0.022 (3)
C30	0.134 (4)	0.151 (5)	0.156 (5)	−0.027 (4)	0.024 (4)	−0.050 (4)
C31	0.0513 (14)	0.0569 (15)	0.0727 (17)	−0.0012 (12)	0.0051 (12)	−0.0202 (13)
C32	0.0625 (16)	0.0614 (17)	0.090 (2)	−0.0149 (14)	0.0090 (14)	−0.0191 (15)
C33	0.0642 (17)	0.0661 (18)	0.0824 (19)	−0.0119 (14)	−0.0029 (14)	−0.0271 (16)
C34	0.0486 (13)	0.0603 (16)	0.0735 (17)	0.0015 (12)	−0.0080 (12)	−0.0123 (13)
C35	0.0581 (15)	0.0654 (17)	0.0741 (18)	−0.0115 (13)	0.0024 (13)	−0.0150 (14)
C36	0.0581 (15)	0.0647 (17)	0.0783 (18)	−0.0159 (13)	0.0010 (13)	−0.0233 (15)
C37	0.0648 (17)	0.0702 (18)	0.0710 (17)	0.0051 (14)	−0.0095 (14)	−0.0148 (15)
C38	0.121 (3)	0.140 (4)	0.085 (3)	−0.018 (3)	0.033 (2)	−0.023 (3)
C39	0.187 (6)	0.127 (4)	0.095 (3)	−0.052 (4)	0.030 (3)	−0.017 (3)
C40	0.302 (11)	0.118 (5)	0.134 (5)	0.005 (6)	−0.015 (6)	−0.009 (4)
C41	0.0483 (13)	0.0645 (16)	0.0651 (16)	0.0056 (12)	0.0138 (11)	−0.0068 (13)
C42	0.0695 (17)	0.0688 (18)	0.0746 (18)	0.0117 (15)	0.0074 (14)	0.0016 (15)
C43	0.0735 (19)	0.087 (2)	0.0670 (18)	0.0105 (16)	−0.0003 (14)	−0.0033 (16)
C44	0.0651 (17)	0.079 (2)	0.0649 (17)	−0.0006 (15)	0.0095 (13)	−0.0102 (15)
C45	0.080 (2)	0.0605 (17)	0.0754 (19)	0.0045 (15)	0.0076 (15)	0.0004 (15)
C46	0.0680 (17)	0.0689 (18)	0.0644 (16)	0.0054 (14)	0.0008 (13)	−0.0014 (14)
C47	0.098 (3)	0.089 (3)	0.083 (2)	−0.005 (2)	0.0072 (19)	−0.016 (2)
C48	0.162 (5)	0.101 (3)	0.117 (4)	−0.042 (3)	−0.008 (3)	−0.030 (3)
C49	0.260 (8)	0.108 (4)	0.111 (4)	−0.075 (5)	0.018 (4)	−0.017 (3)
C50	0.344 (11)	0.088 (4)	0.128 (5)	−0.031 (5)	0.005 (5)	−0.026 (3)
C51	0.0522 (14)	0.0610 (16)	0.0661 (16)	0.0090 (12)	−0.0119 (12)	−0.0015 (13)
C52	0.0741 (18)	0.0656 (18)	0.0782 (19)	−0.0167 (15)	−0.0116 (15)	0.0003 (15)
C53	0.098 (2)	0.0636 (18)	0.0709 (19)	−0.0127 (17)	−0.0123 (16)	−0.0090 (15)
C54	0.0754 (18)	0.0580 (16)	0.0729 (18)	0.0040 (14)	−0.0105 (14)	0.0032 (14)
C55	0.0800 (19)	0.0546 (16)	0.081 (2)	−0.0059 (14)	−0.0089 (15)	0.0043 (15)
C56	0.0765 (18)	0.0522 (15)	0.079 (2)	−0.0031 (14)	−0.0151 (15)	−0.0060 (14)
C57	0.105 (3)	0.069 (2)	0.085 (2)	−0.0004 (19)	0.0067 (19)	−0.0038 (18)
C58	0.163 (4)	0.106 (3)	0.089 (3)	−0.024 (3)	0.028 (3)	0.012 (2)
C59	0.203 (7)	0.112 (9)	0.072 (4)	−0.035 (10)	0.005 (4)	0.003 (7)
C59A	0.203 (7)	0.112 (9)	0.072 (4)	−0.035 (10)	0.005 (4)	0.003 (7)
C60	0.195 (9)	0.148 (10)	0.094 (6)	0.013 (10)	0.008 (5)	0.003 (7)
C60A	0.195 (9)	0.148 (10)	0.094 (6)	0.013 (10)	0.008 (5)	0.003 (7)
N1	0.0539 (12)	0.0540 (13)	0.0753 (14)	0.0042 (10)	0.0040 (10)	−0.0087 (11)
N2	0.0466 (11)	0.0671 (15)	0.0793 (15)	−0.0016 (10)	−0.0028 (10)	−0.0134 (12)
N3	0.0499 (11)	0.0504 (12)	0.0571 (11)	−0.0024 (9)	0.0019 (9)	−0.0003 (9)
O1	0.0656 (11)	0.0577 (11)	0.0623 (11)	−0.0030 (9)	−0.0058 (8)	0.0030 (9)
O2	0.173 (3)	0.117 (2)	0.0617 (15)	−0.011 (2)	−0.0181 (15)	−0.0168 (14)
O3	0.133 (2)	0.0871 (17)	0.0714 (14)	−0.0286 (15)	−0.0079 (13)	−0.0201 (13)
O4	0.0448 (9)	0.0748 (12)	0.0687 (11)	0.0009 (8)	0.0026 (8)	−0.0096 (9)
O5	0.182 (3)	0.119 (3)	0.096 (2)	0.020 (2)	0.023 (2)	−0.0315 (19)
O6	0.271 (5)	0.108 (2)	0.0799 (18)	0.002 (3)	0.040 (2)	0.0021 (18)
O7	0.0786 (13)	0.0614 (12)	0.0806 (13)	−0.0176 (10)	0.0198 (10)	−0.0163 (10)
O8	0.1113 (19)	0.111 (2)	0.0703 (14)	0.0221 (15)	−0.0137 (13)	0.0055 (13)
O9	0.0830 (14)	0.0920 (16)	0.0710 (13)	0.0067 (12)	0.0046 (10)	−0.0146 (12)
O10	0.0726 (12)	0.0652 (12)	0.0821 (13)	−0.0170 (10)	0.0149 (10)	−0.0229 (10)
O11	0.1110 (18)	0.0880 (17)	0.0777 (14)	−0.0159 (14)	−0.0061 (12)	−0.0286 (13)
O12	0.0950 (16)	0.1080 (19)	0.0796 (14)	−0.0235 (15)	0.0133 (12)	−0.0245 (13)
O13	0.0559 (10)	0.0619 (11)	0.0851 (13)	0.0016 (9)	0.0144 (9)	−0.0115 (10)
O14	0.206 (4)	0.120 (3)	0.102 (2)	−0.014 (2)	−0.045 (2)	−0.0251 (19)
O15	0.119 (2)	0.0800 (16)	0.0997 (18)	−0.0174 (15)	0.0022 (15)	−0.0193 (14)
O16	0.0643 (11)	0.0674 (12)	0.0723 (12)	0.0169 (9)	−0.0042 (9)	−0.0010 (10)
O17	0.235 (4)	0.0822 (19)	0.102 (2)	−0.016 (2)	0.045 (2)	−0.0106 (17)
O18	0.151 (2)	0.0760 (16)	0.0851 (16)	−0.0177 (16)	0.0050 (15)	0.0073 (13)
P1	0.0452 (3)	0.0521 (4)	0.0588 (4)	0.0009 (3)	0.0015 (3)	−0.0048 (3)
P2	0.0542 (4)	0.0528 (4)	0.0691 (4)	−0.0064 (3)	0.0090 (3)	−0.0149 (3)
P3	0.0467 (3)	0.0549 (4)	0.0635 (4)	0.0036 (3)	0.0016 (3)	−0.0064 (3)

### Geometric parameters (Å, º)

**Table:**

C1—C2	1.379 (4)	C33—C34	1.369 (4)
C1—C6	1.380 (4)	C34—C35	1.381 (4)
C1—O1	1.398 (3)	C34—C37	1.489 (4)
C2—H2	0.9300	C35—H35	0.9300
C2—C3	1.367 (4)	C35—C36	1.384 (4)
C3—H3	0.9300	C36—H36	0.9300
C3—C4	1.380 (4)	C37—O11	1.198 (3)
C4—C5	1.392 (4)	C37—O12	1.331 (4)
C4—C7	1.486 (4)	C38—H38A	0.9700
C5—H5	0.9300	C38—H38B	0.9700
C5—C6	1.380 (4)	C38—C39	1.468 (7)
C6—H6	0.9300	C38—O12	1.438 (4)
C7—O2	1.207 (4)	C39—H39	0.9300
C7—O3	1.322 (4)	C39—C40	1.255 (8)
C8—H8A	0.9700	C40—H40A	0.9300
C8—H8B	0.9700	C40—H40B	0.9300
C8—C9	1.415 (7)	C41—C42	1.369 (4)
C8—O3	1.471 (4)	C41—C46	1.364 (4)
C9—H9	0.9300	C41—O13	1.408 (3)
C9—C10	1.262 (7)	C42—H42	0.9300
C10—H10A	0.9300	C42—C43	1.379 (4)
C10—H10B	0.9300	C43—H43	0.9300
C11—C12	1.375 (4)	C43—C44	1.375 (4)
C11—C16	1.371 (4)	C44—C45	1.393 (4)
C11—O4	1.394 (3)	C44—C47	1.489 (5)
C12—H12	0.9300	C45—H45	0.9300
C12—C13	1.382 (4)	C45—C46	1.384 (4)
C13—H13	0.9300	C46—H46	0.9300
C13—C14	1.379 (4)	C47—O14	1.190 (4)
C14—C15	1.386 (4)	C47—O15	1.330 (5)
C14—C17	1.487 (5)	C48—H48A	0.9700
C15—H15	0.9300	C48—H48B	0.9700
C15—C16	1.381 (4)	C48—C49	1.413 (7)
C16—H16	0.9300	C48—O15	1.469 (4)
C17—O5	1.194 (4)	C49—H49	0.9300
C17—O6	1.297 (5)	C49—C50	1.308 (8)
C18—H18A	0.9700	C50—H50A	0.9300
C18—H18B	0.9700	C50—H50B	0.9300
C18—C19	1.54 (2)	C51—C52	1.371 (4)
C18—O6	1.39 (5)	C51—C56	1.377 (4)
C18A—H18C	0.9700	C51—O16	1.393 (3)
C18A—H18D	0.9700	C52—H52	0.9300
C18A—C19A	1.469 (12)	C52—C53	1.381 (5)
C18A—O6	1.500 (14)	C53—H53	0.9300
C19—H19	0.9300	C53—C54	1.390 (4)
C19—C20	1.358 (19)	C54—C55	1.375 (4)
C19A—H19A	0.9300	C54—C57	1.489 (5)
C19A—C20A	1.192 (12)	C55—H55	0.9300
C20—H20C	0.9300	C55—C56	1.378 (4)
C20—H20D	0.9300	C56—H56	0.9300
C20A—H20A	0.9300	C57—O17	1.197 (4)
C20A—H20B	0.9300	C57—O18	1.317 (4)
C21—C22	1.382 (4)	C58—H58A	0.9700
C21—C26	1.376 (4)	C58—H58B	0.9700
C21—O7	1.408 (3)	C58—H58C	0.9700
C22—H22	0.9300	C58—H58D	0.9700
C22—C23	1.370 (4)	C58—C59	1.448 (14)
C23—H23	0.9300	C58—C59A	1.49 (2)
C23—C24	1.391 (4)	C58—O18	1.460 (5)
C24—C25	1.391 (4)	C59—H59	0.9300
C24—C27	1.488 (4)	C59—C60	1.306 (17)
C25—H25	0.9300	C59A—H59A	0.9300
C25—C26	1.378 (4)	C59A—C60A	1.31 (2)
C26—H26	0.9300	C60—H60A	0.9300
C27—O8	1.194 (4)	C60—H60B	0.9300
C27—O9	1.337 (4)	C60A—H60C	0.9300
C28—H28A	0.9700	C60A—H60D	0.9300
C28—H28B	0.9700	N1—P1	1.580 (2)
C28—C29	1.473 (6)	N1—P2	1.582 (2)
C28—O9	1.451 (4)	N2—P2	1.575 (2)
C29—H29	0.9300	N2—P3	1.579 (2)
C29—C30	1.235 (6)	N3—P1	1.578 (2)
C30—H30A	0.9300	N3—P3	1.585 (2)
C30—H30B	0.9300	O1—P1	1.5833 (19)
C31—C32	1.387 (4)	O4—P1	1.5826 (18)
C31—C36	1.361 (4)	O7—P2	1.591 (2)
C31—O10	1.391 (3)	O10—P2	1.5834 (19)
C32—H32	0.9300	O13—P3	1.579 (2)
C32—C33	1.375 (4)	O16—P3	1.583 (2)
C33—H33	0.9300		
			
C2—C1—C6	121.6 (3)	C35—C36—H36	120.6
C2—C1—O1	115.4 (2)	O11—C37—C34	124.6 (3)
C6—C1—O1	123.0 (2)	O11—C37—O12	123.4 (3)
C1—C2—H2	120.4	O12—C37—C34	111.9 (2)
C3—C2—C1	119.1 (3)	H38A—C38—H38B	107.9
C3—C2—H2	120.4	C39—C38—H38A	109.2
C2—C3—H3	119.5	C39—C38—H38B	109.2
C2—C3—C4	121.0 (3)	O12—C38—H38A	109.2
C4—C3—H3	119.5	O12—C38—H38B	109.2
C3—C4—C5	119.0 (3)	O12—C38—C39	111.9 (4)
C3—C4—C7	119.6 (3)	C38—C39—H39	116.0
C5—C4—C7	121.5 (3)	C40—C39—C38	128.0 (6)
C4—C5—H5	119.6	C40—C39—H39	116.0
C6—C5—C4	120.8 (3)	C39—C40—H40A	120.0
C6—C5—H5	119.6	C39—C40—H40B	120.0
C1—C6—H6	120.8	H40A—C40—H40B	120.0
C5—C6—C1	118.5 (2)	C42—C41—O13	117.4 (3)
C5—C6—H6	120.8	C46—C41—C42	122.3 (3)
O2—C7—C4	123.6 (4)	C46—C41—O13	120.2 (2)
O2—C7—O3	123.2 (3)	C41—C42—H42	120.6
O3—C7—C4	113.1 (3)	C41—C42—C43	118.8 (3)
H8A—C8—H8B	108.3	C43—C42—H42	120.6
C9—C8—H8A	110.0	C42—C43—H43	119.5
C9—C8—H8B	110.0	C44—C43—C42	120.9 (3)
C9—C8—O3	108.7 (4)	C44—C43—H43	119.5
O3—C8—H8A	110.0	C43—C44—C45	118.8 (3)
O3—C8—H8B	110.0	C43—C44—C47	118.0 (3)
C8—C9—H9	116.6	C45—C44—C47	123.2 (3)
C10—C9—C8	126.8 (7)	C44—C45—H45	119.6
C10—C9—H9	116.6	C46—C45—C44	120.7 (3)
C9—C10—H10A	120.0	C46—C45—H45	119.6
C9—C10—H10B	120.0	C41—C46—C45	118.4 (3)
H10A—C10—H10B	120.0	C41—C46—H46	120.8
C12—C11—O4	118.2 (3)	C45—C46—H46	120.8
C16—C11—C12	121.5 (3)	O14—C47—C44	124.5 (4)
C16—C11—O4	120.2 (2)	O14—C47—O15	123.3 (4)
C11—C12—H12	120.7	O15—C47—C44	112.2 (3)
C11—C12—C13	118.6 (3)	H48A—C48—H48B	108.6
C13—C12—H12	120.7	C49—C48—H48A	110.3
C12—C13—H13	119.4	C49—C48—H48B	110.3
C14—C13—C12	121.2 (3)	C49—C48—O15	107.1 (4)
C14—C13—H13	119.4	O15—C48—H48A	110.3
C13—C14—C15	118.8 (3)	O15—C48—H48B	110.3
C13—C14—C17	118.5 (3)	C48—C49—H49	117.0
C15—C14—C17	122.7 (3)	C50—C49—C48	126.1 (6)
C14—C15—H15	119.7	C50—C49—H49	117.0
C16—C15—C14	120.6 (3)	C49—C50—H50A	120.0
C16—C15—H15	119.7	C49—C50—H50B	120.0
C11—C16—C15	119.3 (3)	H50A—C50—H50B	120.0
C11—C16—H16	120.4	C52—C51—C56	121.3 (3)
C15—C16—H16	120.4	C52—C51—O16	120.1 (3)
O5—C17—C14	124.8 (4)	C56—C51—O16	118.4 (3)
O5—C17—O6	122.7 (4)	C51—C52—H52	120.6
O6—C17—C14	112.5 (3)	C51—C52—C53	118.7 (3)
H18A—C18—H18B	108.2	C53—C52—H52	120.6
C19—C18—H18A	109.7	C52—C53—H53	119.7
C19—C18—H18B	109.7	C52—C53—C54	120.7 (3)
O6—C18—H18A	109.7	C54—C53—H53	119.7
O6—C18—H18B	109.7	C53—C54—C57	118.8 (3)
O6—C18—C19	110 (4)	C55—C54—C53	119.5 (3)
H18C—C18A—H18D	108.5	C55—C54—C57	121.7 (3)
C19A—C18A—H18C	110.3	C54—C55—H55	119.9
C19A—C18A—H18D	110.3	C54—C55—C56	120.2 (3)
C19A—C18A—O6	107.3 (8)	C56—C55—H55	119.9
O6—C18A—H18C	110.3	C51—C56—C55	119.6 (3)
O6—C18A—H18D	110.3	C51—C56—H56	120.2
C18—C19—H19	119.2	C55—C56—H56	120.2
C20—C19—C18	122 (3)	O17—C57—C54	123.5 (4)
C20—C19—H19	119.2	O17—C57—O18	123.2 (4)
C18A—C19A—H19A	116.5	O18—C57—C54	113.2 (3)
C20A—C19A—C18A	127.1 (12)	H58A—C58—H58B	107.8
C20A—C19A—H19A	116.5	H58C—C58—H58D	108.3
C19—C20—H20C	120.0	C59—C58—H58A	109.0
C19—C20—H20D	120.0	C59—C58—H58B	109.0
H20C—C20—H20D	120.0	C59—C58—O18	112.8 (8)
C19A—C20A—H20A	120.0	C59A—C58—H58C	109.9
C19A—C20A—H20B	120.0	C59A—C58—H58D	109.9
H20A—C20A—H20B	120.0	O18—C58—H58A	109.0
C22—C21—O7	119.1 (3)	O18—C58—H58B	109.0
C26—C21—C22	121.9 (3)	O18—C58—H58C	109.9
C26—C21—O7	119.0 (3)	O18—C58—H58D	109.9
C21—C22—H22	120.5	O18—C58—C59A	108.9 (9)
C23—C22—C21	119.1 (3)	C58—C59—H59	122.2
C23—C22—H22	120.5	C60—C59—C58	115.6 (13)
C22—C23—H23	119.8	C60—C59—H59	122.2
C22—C23—C24	120.4 (3)	C58—C59A—H59A	116.6
C24—C23—H23	119.8	C60A—C59A—C58	126.9 (17)
C23—C24—C25	119.5 (3)	C60A—C59A—H59A	116.6
C23—C24—C27	118.4 (3)	C59—C60—H60A	120.0
C25—C24—C27	122.1 (3)	C59—C60—H60B	120.0
C24—C25—H25	119.8	H60A—C60—H60B	120.0
C26—C25—C24	120.4 (3)	C59A—C60A—H60C	120.0
C26—C25—H25	119.8	C59A—C60A—H60D	120.0
C21—C26—C25	118.8 (3)	H60C—C60A—H60D	120.0
C21—C26—H26	120.6	P1—N1—P2	121.83 (14)
C25—C26—H26	120.6	P2—N2—P3	121.66 (14)
O8—C27—C24	124.6 (3)	P1—N3—P3	120.99 (13)
O8—C27—O9	123.0 (3)	C1—O1—P1	127.46 (16)
O9—C27—C24	112.3 (3)	C7—O3—C8	116.3 (3)
H28A—C28—H28B	108.2	C11—O4—P1	122.43 (15)
C29—C28—H28A	109.8	C17—O6—C18	132.7 (17)
C29—C28—H28B	109.8	C17—O6—C18A	112.4 (6)
O9—C28—H28A	109.8	C21—O7—P2	119.14 (17)
O9—C28—H28B	109.8	C27—O9—C28	116.7 (3)
O9—C28—C29	109.4 (3)	C31—O10—P2	129.42 (18)
C28—C29—H29	115.8	C37—O12—C38	116.9 (3)
C30—C29—C28	128.3 (6)	C41—O13—P3	123.69 (17)
C30—C29—H29	115.8	C47—O15—C48	114.9 (3)
C29—C30—H30A	120.0	C51—O16—P3	124.73 (17)
C29—C30—H30B	120.0	C57—O18—C58	117.2 (3)
H30A—C30—H30B	120.0	N1—P1—O1	106.67 (12)
C32—C31—O10	114.6 (3)	N1—P1—O4	110.10 (11)
C36—C31—C32	121.3 (3)	N3—P1—N1	117.38 (11)
C36—C31—O10	124.0 (2)	N3—P1—O1	111.80 (11)
C31—C32—H32	120.6	N3—P1—O4	110.71 (11)
C33—C32—C31	118.8 (3)	O4—P1—O1	98.48 (10)
C33—C32—H32	120.6	N1—P2—O7	110.08 (13)
C32—C33—H33	119.5	N1—P2—O10	111.51 (11)
C34—C33—C32	121.0 (3)	N2—P2—N1	116.65 (12)
C34—C33—H33	119.5	N2—P2—O7	111.92 (13)
C33—C34—C35	119.2 (3)	N2—P2—O10	111.12 (12)
C33—C34—C37	118.7 (3)	O10—P2—O7	93.16 (11)
C35—C34—C37	122.1 (3)	N2—P3—N3	117.08 (12)
C34—C35—H35	119.6	N2—P3—O16	111.34 (12)
C34—C35—C36	120.9 (3)	O13—P3—N2	106.32 (12)
C36—C35—H35	119.6	O13—P3—N3	112.30 (11)
C31—C36—C35	118.8 (3)	O13—P3—O16	99.19 (11)
C31—C36—H36	120.6	O16—P3—N3	109.16 (11)
			
C1—C2—C3—C4	0.6 (4)	C41—O13—P3—O16	−77.2 (2)
C1—O1—P1—N1	−168.6 (2)	C42—C41—C46—C45	2.0 (4)
C1—O1—P1—N3	−39.0 (2)	C42—C41—O13—P3	−97.7 (3)
C1—O1—P1—O4	77.4 (2)	C42—C43—C44—C45	1.4 (5)
C2—C1—C6—C5	−2.5 (4)	C42—C43—C44—C47	−178.2 (3)
C2—C1—O1—P1	156.6 (2)	C43—C44—C45—C46	−0.8 (5)
C2—C3—C4—C5	−2.4 (4)	C43—C44—C47—O14	−10.1 (6)
C2—C3—C4—C7	177.0 (3)	C43—C44—C47—O15	170.1 (3)
C3—C4—C5—C6	1.8 (4)	C44—C45—C46—C41	−0.8 (5)
C3—C4—C7—O2	8.8 (5)	C44—C47—O15—C48	−179.6 (3)
C3—C4—C7—O3	−169.0 (3)	C45—C44—C47—O14	170.3 (4)
C4—C5—C6—C1	0.6 (4)	C45—C44—C47—O15	−9.5 (5)
C4—C7—O3—C8	177.7 (4)	C46—C41—C42—C43	−1.4 (4)
C5—C4—C7—O2	−171.7 (3)	C46—C41—O13—P3	86.5 (3)
C5—C4—C7—O3	10.4 (4)	C47—C44—C45—C46	178.8 (3)
C6—C1—C2—C3	1.9 (4)	C49—C48—O15—C47	168.2 (5)
C6—C1—O1—P1	−24.7 (3)	C51—C52—C53—C54	−1.2 (5)
C7—C4—C5—C6	−177.7 (3)	C51—O16—P3—N2	−72.4 (2)
C9—C8—O3—C7	−177.4 (4)	C51—O16—P3—N3	58.4 (2)
C11—C12—C13—C14	0.3 (4)	C51—O16—P3—O13	176.0 (2)
C11—O4—P1—N1	60.0 (2)	C52—C51—C56—C55	−0.1 (4)
C11—O4—P1—N3	−71.5 (2)	C52—C51—O16—P3	68.2 (3)
C11—O4—P1—O1	171.3 (2)	C52—C53—C54—C55	0.4 (5)
C12—C11—C16—C15	0.4 (4)	C52—C53—C54—C57	−178.4 (3)
C12—C11—O4—P1	−102.4 (2)	C53—C54—C55—C56	0.6 (5)
C12—C13—C14—C15	−0.6 (4)	C53—C54—C57—O17	−17.3 (6)
C12—C13—C14—C17	178.8 (3)	C53—C54—C57—O18	161.2 (3)
C13—C14—C15—C16	0.8 (4)	C54—C55—C56—C51	−0.7 (4)
C13—C14—C17—O5	5.2 (5)	C54—C57—O18—C58	−177.3 (3)
C13—C14—C17—O6	−174.2 (3)	C55—C54—C57—O17	164.0 (4)
C14—C15—C16—C11	−0.7 (5)	C55—C54—C57—O18	−17.6 (5)
C14—C17—O6—C18	−171 (3)	C56—C51—C52—C53	1.0 (4)
C14—C17—O6—C18A	176.3 (9)	C56—C51—O16—P3	−116.8 (3)
C15—C14—C17—O5	−175.3 (4)	C57—C54—C55—C56	179.3 (3)
C15—C14—C17—O6	5.2 (5)	C59—C58—O18—C57	72.7 (7)
C16—C11—C12—C13	−0.2 (4)	C59A—C58—O18—C57	102.0 (7)
C16—C11—O4—P1	81.5 (3)	O1—C1—C2—C3	−179.4 (2)
C17—C14—C15—C16	−178.6 (3)	O1—C1—C6—C5	178.8 (2)
C19—C18—O6—C17	−110 (3)	O2—C7—O3—C8	−0.2 (6)
C19A—C18A—O6—C17	−153.1 (10)	O3—C8—C9—C10	137.7 (6)
C21—C22—C23—C24	−1.8 (4)	O4—C11—C12—C13	−176.3 (2)
C21—O7—P2—N1	61.4 (2)	O4—C11—C16—C15	176.4 (3)
C21—O7—P2—N2	−70.0 (2)	O5—C17—O6—C18	10 (3)
C21—O7—P2—O10	175.7 (2)	O5—C17—O6—C18A	−3.1 (10)
C22—C21—C26—C25	1.3 (5)	O6—C18—C19—C20	−116 (4)
C22—C21—O7—P2	−93.3 (3)	O6—C18A—C19A—C20A	−11 (2)
C22—C23—C24—C25	2.9 (4)	O7—C21—C22—C23	−179.4 (2)
C22—C23—C24—C27	−175.2 (3)	O7—C21—C26—C25	−179.6 (3)
C23—C24—C25—C26	−1.8 (4)	O8—C27—O9—C28	6.5 (5)
C23—C24—C27—O8	0.6 (5)	O9—C28—C29—C30	−109.5 (5)
C23—C24—C27—O9	178.1 (3)	O10—C31—C32—C33	−178.2 (3)
C24—C25—C26—C21	−0.2 (4)	O10—C31—C36—C35	178.5 (3)
C24—C27—O9—C28	−171.1 (3)	O11—C37—O12—C38	1.3 (5)
C25—C24—C27—O8	−177.4 (3)	O12—C38—C39—C40	5.4 (9)
C25—C24—C27—O9	0.1 (4)	O13—C41—C42—C43	−177.1 (3)
C26—C21—C22—C23	−0.3 (4)	O13—C41—C46—C45	177.6 (2)
C26—C21—O7—P2	87.6 (3)	O14—C47—O15—C48	0.6 (6)
C27—C24—C25—C26	176.1 (3)	O15—C48—C49—C50	130.4 (7)
C29—C28—O9—C27	85.8 (4)	O16—C51—C52—C53	175.9 (3)
C31—C32—C33—C34	−0.2 (5)	O16—C51—C56—C55	−175.0 (2)
C31—O10—P2—N1	−66.2 (3)	O17—C57—O18—C58	1.2 (6)
C31—O10—P2—N2	65.8 (3)	O18—C58—C59—C60	113.3 (14)
C31—O10—P2—O7	−179.2 (2)	O18—C58—C59A—C60A	−124.6 (19)
C32—C31—C36—C35	−0.2 (5)	P1—N1—P2—N2	15.8 (2)
C32—C31—O10—P2	−169.9 (2)	P1—N1—P2—O7	−113.10 (17)
C32—C33—C34—C35	−0.7 (5)	P1—N1—P2—O10	144.95 (16)
C32—C33—C34—C37	177.4 (3)	P1—N3—P3—N2	−17.9 (2)
C33—C34—C35—C36	1.2 (4)	P1—N3—P3—O13	105.55 (16)
C33—C34—C37—O11	4.2 (5)	P1—N3—P3—O16	−145.47 (14)
C33—C34—C37—O12	−172.7 (3)	P2—N1—P1—N3	−15.0 (2)
C34—C35—C36—C31	−0.7 (5)	P2—N1—P1—O1	111.30 (17)
C34—C37—O12—C38	178.3 (3)	P2—N1—P1—O4	−142.83 (16)
C35—C34—C37—O11	−177.7 (3)	P2—N2—P3—N3	19.0 (2)
C35—C34—C37—O12	5.4 (4)	P2—N2—P3—O13	−107.40 (18)
C36—C31—C32—C33	0.7 (5)	P2—N2—P3—O16	145.56 (16)
C36—C31—O10—P2	11.3 (4)	P3—N2—P2—N1	−17.9 (2)
C37—C34—C35—C36	−176.9 (3)	P3—N2—P2—O7	110.11 (18)
C39—C38—O12—C37	93.4 (5)	P3—N2—P2—O10	−147.22 (16)
C41—C42—C43—C44	−0.3 (5)	P3—N3—P1—N1	15.9 (2)
C41—O13—P3—N2	167.2 (2)	P3—N3—P1—O1	−107.78 (15)
C41—O13—P3—N3	38.0 (2)	P3—N3—P1—O4	143.49 (14)
