# Supplementary figures and images for: Crystal structure of hexa­prop-2-en-1-yl 4,4′,4′′,4′′′,4′′′′,4′′′′′-[1,3,5,2λ5,4λ5,6λ5-tri­aza­triphosphinine-2,2,4,4,6,6-hexa­yl­hexa­kis­(­oxy)]hexa­benzoate
Source: Acta Crystallogr E Crystallogr Commun. 2015 Nov 18;71(Pt 12):o955–6. doi: 10.1107/S2056989015021301 (PMC4719920; doi:10.1107/S2056989015021301)

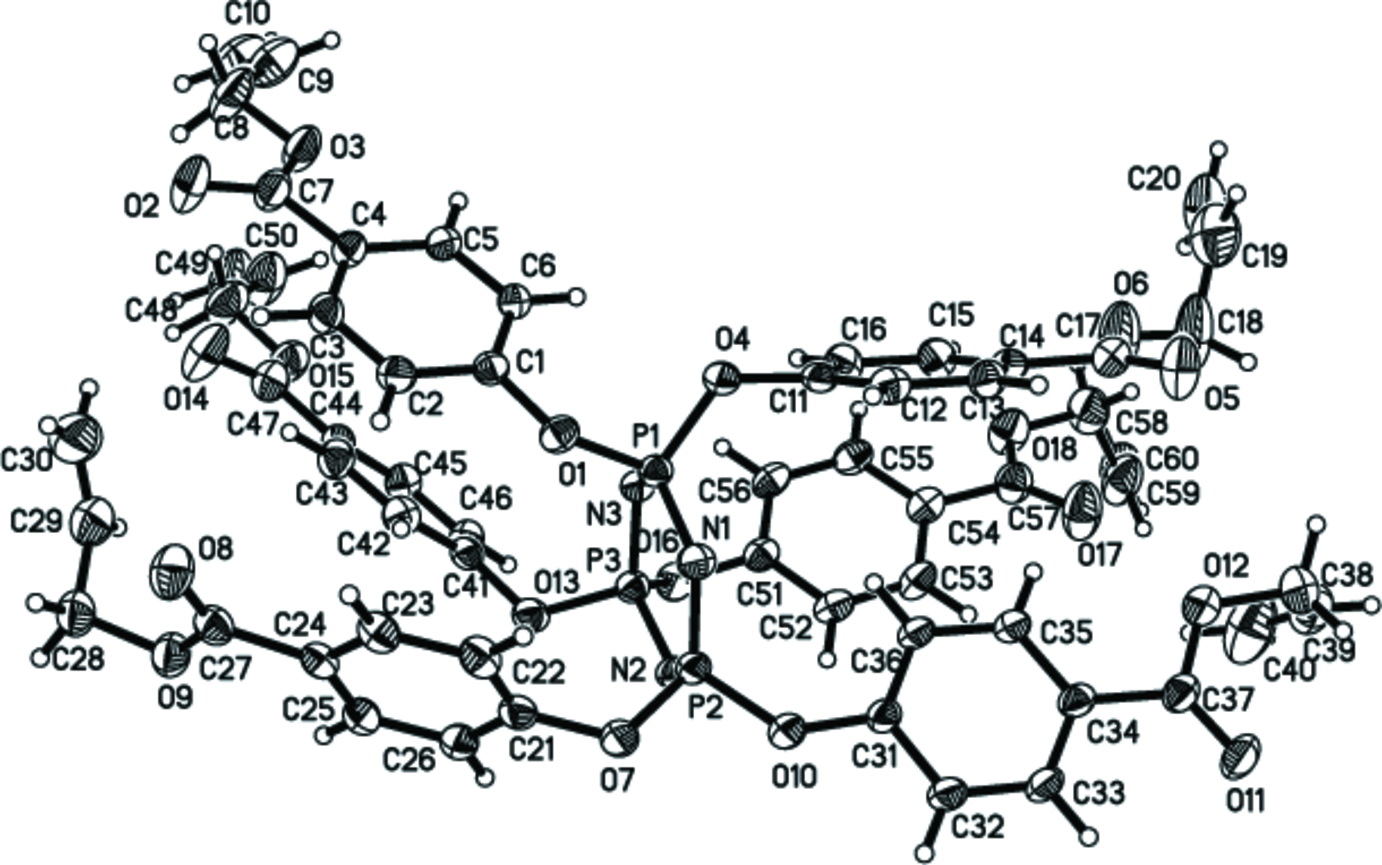

Supplement: Supplementary file 4 [file e-71-0o955-fig1.tif]
